# Psychotropic Management in Cotard Syndrome: Case Reports Supporting Dual Medication Management

**DOI:** 10.1155/2024/7630713

**Published:** 2024-04-09

**Authors:** Adam J. Fusick, Chemar Davis, Steven Gunther, Cory Klippel, Gregory Sullivan

**Affiliations:** ^1^Mental Health and Behavioral Sciences Service, Department of Psychiatry and Behavioral Neurosciences, James A Haley Veterans Hospital, Tampa, Florida, USA; ^2^College of Medicine, University of South Florida, Tampa, Florida, USA

## Abstract

Cotard syndrome is a rare presentation where patients present with nihilistic thoughts of dying or already being dead. These delusions manifest from either a medical or psychiatric etiology and can be difficult to treat. Recently Couto and Gonçalves purposed that treatment should include an atypical antipsychotic alone or in combination with either a mood stabilizer or antidepressant. Here the authors advocate for a more specific but well-known psychotropic regimen, namely the combination of olanzapine and fluoxetine. We conducted a literature review and of 246 papers identified, only three reported using a combination of fluoxetine and olanzapine with many of them having limited or confounding information that make it difficult for us to comment on the historically efficacy of this medication combination. Therefore, the authors provide two case examples of patients being treated successfully with olanzapine and fluoxetine. One, a 66-year-old male veteran and another 76-year-old male veteran. Both of these cases hold significance as the patient's psychotic depression was so severe as to warrant ECT as a possible treatment. In both cases, this medication combination was able to avoid the procedure. Overall, with the addition of our cases and the sparse information available in the literature, we propose the combination of fluoxetine and olanzapine as an effective Cotard syndrome treatment.

## 1. Introduction

Since its first meticulous description by Jules Cotard in 1880, Cotard's syndrome has posed a unique treatment challenge given its various etiologies and spectrum of symptom severity [1]. This syndrome is characterized by a patients' nihilistic denial of one-self that can range in intensity from parts of one's body to the entire body or even extending to one's own existence [2–4]. Cotard's syndrome remains an enigmatic concept in modern psychiatric understanding and is not recognized as a distinct psychiatric disorder in the current DSM-5 [5]. Cotard's syndrome, therefore, can be associated with various underlying pathologies, ranging from neurological conditions like cerebral atrophy and temporal lobe epilepsy to psychiatric disorders such as major depressive disorder (MDD) [5–7]. Particularly, noteworthy is this delusions manifestation in MDD with psychotic features, which can present as severe depressive delusions, including profound feelings of guilt or worthlessness that distort self-perception to the point of self-perceived nonexistence or death [4, 8]. This distorted view may cause patients to cease eating or have an increased tendency toward self-mutilation or suicidal behavior [8].

Given the complex clinical profile and the severe risk of self-harm and suicide associated with Cotard's syndrome, there is an imperative need for effective treatment strategies [8]. Couto and Moreira Goncalves [9] provided guidance on this issue and proposed that the initial treatment approach should include an atypical antipsychotic alone or in combination with either a mood stabilizer or antidepressant. While these recommendations are helpful, they stop short of suggesting specific medications. While conventional pharmacotherapy has shown promise, recent research also emphasizes the role of electroconvulsive therapy (ECT) as a significant treatment option, especially in cases unresponsive to pharmacotherapy [10, 11]. One pivotal Japanese meta-analysis of 130 Cotard's syndrome cases showed a 21.6% effectiveness rate for ECT, underscoring its efficacy in treatment-resistant cases and advocating its consideration as a primary treatment option [12]. These findings highlight the need for a tailored approach in pharmacotherapy with a nuanced understanding of drug responses in Cotard's syndrome [9].

Our case series focuses on two American veterans with Cotard's syndrome, who exhibited positive responses to a combination of olanzapine and fluoxetine, obviating the immediate need for ECT. This narrative illustrates the efficacy of this therapeutic strategy, one that is commonly used in severe MDD with psychotic features, in resolving severe psychotic symptoms even before ECT could be court approved [13]. These cases also exemplify the potential of this pharmacological approach as an effective alternative when ECT is delayed or inaccessible and thus advances our understanding of Cotard's syndrome treatment.

## 2. Case Summary 1

Mr. A, a 66-year-old Caucasian male, recently unemployed, without prior psychiatric history but with a past medical history of diabetes, hypertension, hyperlipidemia, benign prostatic hyperplasia, and renal calculi. In the 6 days prior to admission, the patient exhibited notable behavioral changes, including nihilistic comments and explicit thoughts of self-harm that were accompanied by poor sleep, depressive mood, and nonspecific visual hallucinations. He was brought into the emergency department for these thoughts and depressed mood he was incidentally found to be SARS-CoV-2 positive. He was admitted to the medical floor for isolation.

While on the medical floor, initial workup included a negative urine drug screening and an unremarkable cranial CT scan. EEG was also negative. The patient met the criteria for MDD with psychotic features. Evaluation at this time was remarkable for marked personal hygiene neglect, social withdrawal, sadness of mood, diminished interest in almost all activities, anhedonia, ideas of worthlessness, hopelessness, and guilt. He also endorsed poor sleep and no appetite despite refusing to eat since being admitted to the hospital. Patient also endorsed experiencing some anxiety secondary to his nihilism which presented as a belief that he was already dead and that his insides were poisonous to others. This belief was so strong that the patient was afraid to urinate or defecate thinking he would kill the entire hospital by doing so. Once cleared from COVID-19, he was transferred to the inpatient psychiatric floor.

The treatment team felt ECT was necessary; however, Florida law and hospital policy require two separate court hearings before initiation. Pharmacotherapy was expediently started to address his deteriorating condition with a combination of olanzapine and fluoxetine. Olanzapine was started at 10 mg a day while fluoxetine was started at 20 mg a day and quickly titrated up to 40 mg daily. A significant improvement in mood, daily functioning, insight, and judgment was observed within a week of psychotropic initiation and thus obviating the need for ECT. Within 4 weeks of initiating this treatment strategy, the patient was discharged from the inpatient unit.

Interestingly, postdischarge, the patient was monitored monthly and after 15 months his outpatient psychiatrist attempted to reduce his psychotropics at the patient's request. Soon after discontinuation of olanzapine along with the reduction of fluoxetine, the patient experienced a recurrence of symptoms which prompted another admission to our inpatient psychiatry ward. The patient was restarted on olanzapine and titrated back up on fluoxetine which abated his Cotard symptoms. He endorsed not wanting to attempt a decrease of his medications in the future as well as not experiencing any side effects to this combination.

## 3. Case Summary 2

Mr. B, 76-year-old Caucasian male with no prior psychiatric episodes but an array of medical conditions, including paroxysmal A-fib, GERD, BPH, OSA, CAD, HTN, and IDDM2 presented to the hospital with mild neurocognitive disorder, anxiety, weight loss, and mourning the recent passing of two children. He was initially assessed with concerns for poor sleep, food refusal, depression, and delusions that began 10 days prior to presenting to the hospital. Initial workup included a CT Head, MRI head with/without contrast, EEG, CT angiogram, and urine drug screening that did not yield any significant findings. Collateral information detailed a marked deterioration of his IADLs and ADLs over the weeks leading to admission. Uniquely, the patient developed an interest in Egyptology, rooted in a “curse” he believed he received during a visit to an Egyptian burial site 2 years ago, which he firmly attributed to his current predicament.

During hospitalization, his condition was characterized by increased social withdrawal, hopelessness, loss of interest in activities, irritability, anhedonia, loss of energy, and insomnia. His nihilistic delusions intensified to include a belief of imminent divine punishment. Constant persuasion was necessary to maintain his oral intake. A diagnosis of MDD with psychotic features was made. Again, not wanting to delay care until ECT was approved, a therapeutic strategy of combined olanzapine and fluoxetine was implemented. Olanzapine was started at 10 mg daily, and fluoxetine was started at 20 mg daily. Within 3 weeks, a complete resolution of symptoms was achieved, and the patient was discharged home. Following discharge, the patient was monitored closely and has remained on this regimen with no concerns for side effects and no return of symptoms.

## 4. Discussion

### 4.1. Review of Cotard's Syndrome Pharmacological and Neuromodulation Treatment Strategies

The compilation of case studies in this report underscores the potential utility of an integrative pharmacotherapeutic strategy utilizing olanzapine and fluoxetine for managing Cotard's syndrome. This approach aligns with the recent recommendations of Couto and Moreira Goncalves [9], but expands on these by suggesting a specific first-line treatment option [9]. This combination of psychotropics is not arbitrary; it stems from a recognition of the effectiveness of this drug combination in treating a range of psychiatric pathologies beyond Cotard's syndrome.

Olanzapine has demonstrated significant efficacy in managing a spectrum of psychiatric conditions [14]. Its pharmacological profile reveals a high affinity for dopamine D2 and serotonin 5-HT2A receptors [13]. This wide-spectrum receptor engagement suggests that olanzapine can modulate diverse neural pathways involved in psychosis, which is critical in addressing both positive and negative symptoms of psychotic disorders [14]. FDA approved for conditions like schizophrenia and bipolar disorder, olanzapine's effectiveness has been observed in other psychiatric disorders as well, making it a versatile drug in psychiatric practice [4, 13, 14]. Its role in treating mood disorders, particularly when psychotic features are present, has been increasingly recognized, offering a promising avenue for managing complex cases like Cotard's syndrome [4].

Fluoxetine, on the other hand, is primarily known for its role in treating MDD [13]. Its mechanism of action involves the inhibition of the serotonin reuptake transporter, but its therapeutic action extends beyond that of simple serotonin reuptake inhibition. Its modulation of the sigma-1 receptor, involved in intracellular calcium signaling and mitochondrial function, brings an additional dimension to its therapeutic profile. The sigma-1 receptor activation is linked to neuroplasticity and neuroprotection [13]. This aspect of fluoxetine's action is particularly relevant in the context of MDD with psychotic features, where neuroplastic changes and neuroprotection are crucial for effective treatment [13].

The combined use of olanzapine and fluoxetine presents a synergistic pharmacotherapeutic strategy that addresses the multifaceted nature of Cotard's syndrome [7]. The mechanism underlying this synergy is explained through complementary actions on various neurotransmitter systems. Olanzapine's antagonistic effect on serotonin 5-HT2A and 5-HT2C receptors may potentiate the serotonergic effects of fluoxetine, leading to enhanced neurotransmitter release in key brain regions like the prefrontal cortex [13]. This interaction could also support the cognitive enhancement and mood elevation often observed in patients treated with this combination [15].

Moreover, the combination of olanzapine and fluoxetine could potentiate neuroplastic changes within cerebral regions implicated in MDD with psychosis. Neuroplasticity, the brain's ability to reorganize itself by forming new neural connections, is a vital component implemented in both psychotic episodes and depressive states [16, 17]. Both psychotropics have been shown to stimulate neurogenesis and increase the expression of brain-derived neurotrophic factor (BDNF) in the hippocampus. The hippocampus, a region often affected in severe depressive states, plays a vital role in memory and emotional responses. Increasing BDNF expression in this region may contribute to the reversal of the hippocampal volume reduction observed in severe depression, offering a path to recovery for patients with complex psychiatric presentations [15].

Lastly, ECT, a well-established treatment modality in psychiatry, remains a significant option for treating Cotard's syndrome [9, 18]. While the exact mechanism of action of ECT is not fully understood, it is known to induce neurochemical changes in the brain that can rapidly alleviate symptoms of severe depression and other psychiatric conditions [19]. In the context of our case studies, a fluoxetine and olanzapine combination negated the necessity for ECT, demonstrating the potency of this pharmacological approach. It is important to recognize, however, that ECT remains a valuable treatment for patients who do not respond to medication. The decision to use ECT is complex and involves considerations of the severity and urgency of symptoms, the patient's medical history, and potential side effects [18]. The application of ECT, while effective, is often limited by accessibility issues and varying legal and ethical regulations across different regions [20–22]. These challenges highlight the importance of having effective pharmacological alternatives, particularly where ECT may not be a feasible option.

### 4.2. Comparative Analysis with Existing Case Reports

The rarity of Cotard's syndrome and the limited use of olanzapine and fluoxetine in combination in clinical practice necessitates a thorough review and comparison with existing literature. Our review revealed just a few instances where this combination was documented. First, Debruyne et al.'s [23] study mentioned that both psychotropics were used in the treatment but did not explicitly mention the concurrent use of olanzapine and fluoxetine, thus leaving a gap in our ability to comment on the effectiveness of this combination in their case comparatively to ours [23]. Another publication, Shaan et al.'s [24] work, which does state that the combination of these psychotropics was used, also deemed the combination ineffective [24]. Notable however, that in this case, the patient had multiple sclerosis (MS) and other complex medical interventions that likely influenced both presentation and treatment outcome. This case also serves to highlight a distinction in our assertion that a fluoxetine–olanzapine combination is likely more effective in Cotard syndrome with a psychogenic etiology. Additionally, further cases also illustrate the need for cautious interpretation and further investigation into the efficacy of specific psychotropics when Cotard's syndrome stem from a medical cause [4, 25, 26].

Lastly, another case presented by Huarcaya-Victoria and Caqui [27] does reinforce the potential utility of the olanzapine and fluoxetine combination but does not provide detailed insights into the severity of the patient's delusions or if the consideration of alternative treatments like ECT were ever considered [27]. This case combined with our cases above does underscore the efficacy of this medication. These variations in clinical presentations and outcomes highlight the unique contribution of our case series in enriching the psychiatric literature and understanding of Cotard's syndrome.

Given these findings, we advocate for a thoughtful reevaluation of primary pharmacotherapy approaches for Cotard's syndrome, emphasizing the use of a medication combination that has been shown to be effective in a broad spectrum of psychiatric disorders, fluoxetine and olanzapine. While in both of our cases, the patients' carry a diagnosis of MDD with psychotic features, this combination could theoretically be useful for other psychiatric disorders that present with Cotard features. Afterall, the combination of olanzapine and fluoxetine has already been shown to be an effective treatment strategy in both schizophrenics and bipolar patients [28, 29]. This consideration becomes particularly useful when realizing that Couto and Moreira Goncalves's [9] analysis reveals that nearly 10% of documented Cotard syndrome cases occur in individuals with who suffer from schizophrenia and an additional 5% are found to be plague by symptoms of bipolar disorder [9].

## 5. Conclusion

Our investigation into Cotard's syndrome underscores the promising potential of an integrated olanzapine and fluoxetine combined treatment. This combination, backed by the individual efficacies of the drugs, suggests a synergistic effect that could yield enhanced therapeutic outcomes in complex condition that is Cotard's syndrome. This concept lays the groundwork for future empirical studies.

The unique insights gained from our case studies, combined with a thorough review of existing literature and an understanding of the condition's pharmacological and psychotherapeutic aspects, pave the way for more effective treatment strategies. Despite dual medications rarely being considered first-line treatment options, our cases along with the trace amounts of supporting studies do highlight effectiveness of this therapeutic approach when Cotard syndrome presents from a psychiatric etiology.

In conclusion, Cotard's syndrome presents a unique challenge to the psychiatric community, necessitating continuous evolution and refinement in both diagnosis and treatment. Understanding this syndrome within a global and cultural context not only deepens our comprehension of its nature but also facilitates the development of more effective treatment strategies. These insights are instrumental in advancing psychiatric practice, ultimately enhancing care for individuals afflicted with this rare but profound disorder and contributing to the broader discourse in psychiatric care and research.

## Data Availability

Data for this manuscript are available upon request.
